# Dissecting GPCR Contributions to Gαo-Dependent Motor Dysfunction in *GNAO1*-Related Disorders Using *Caenorhabditis elegans*

**DOI:** 10.3390/biomedicines14051139

**Published:** 2026-05-18

**Authors:** Martina Di Rocco, Lorenzo Di Rienzo, Francesca Carmen Follo, Manuela D’Alessandro, Serena Galosi, Luca Pannone, Serenella Venanzi, Elia Di Schiavi, Alberto Martire, Jean-Louis Bessereau, Vincenzo Leuzzi, Edoardo Milanetti, Simone Martinelli

**Affiliations:** 1 Department of Oncology and Molecular Medicine, Istituto Superiore di Sanità, 00161 Rome, Italy; martina.dirocco@iss.it (M.D.R.); luca.pannone@iss.it (L.P.); serenella.venanzi@iss.it (S.V.); 2 Center for Life Nano & Neuro Science, Istituto Italiano di Tecnologia, 00161 Rome, Italy; 3 Department of Biochemical Sciences “A. Rossi Fanelli”, Sapienza University, 00185 Rome, Italy; 4 Faculty of Medicine and Pharmacy, University Claude Bernard Lyon 1, MeLiS, CNRS UMR5284, INSERM U1314, 69008 Lyon, France; manuela.dalessandro@univ-lyon1.fr (M.D.); jean-louis.bessereau@univ-lyon1.fr (J.-L.B.); 5 Department of Human Neuroscience, Sapienza University of Rome, 00185 Rome, Italy; serena.galosi@uniroma1.it (S.G.); vincenzo.leuzzi@fondazione.uniroma1.it (V.L.); 6 IBBR Naples Division, Institute of Biosciences and Bioresources, CNR, 80131 Naples, Italy; elia.dischiavi@cnr.it; 7 National Center for Drug Research and Evaluation, Istituto Superiore di Sanità, 00161 Rome, Italy; alberto.martire@iss.it; 8 Department of Physics, Sapienza University of Rome, 00185 Rome, Italy; 9 Link Campus University, 00165 Rome, Italy

**Keywords:** *GNAO1*-related disorders, G protein-coupled receptors, Gαo signaling, *Caenorhabditis elegans*, movement disorders, drug screening, genetic screening

## Abstract

**Background/Objectives**: Pathogenic variants in *GNAO1*, encoding the inhibitory G protein subunit Gαo, cause severe neurodevelopmental disorders that remain largely refractory to pharmacological treatments. Gαo transduces inhibitory signals downstream of multiple G protein-coupled receptors (GPCRs) involved in motor control. Here, we used gene-edited *Caenorhabditis elegans* models carrying *goa-1* variants, the ortholog of *GNAO1*, to investigate GPCR contributions to Gαo-dependent locomotor phenotypes. **Methods**: We combined pharmacological screening of dopamine- and cannabinoid-targeting ligands in *goa-1* mutants with structural analysis of ligand-binding pocket conservation and genetic perturbation of receptor function using RNAi and knockout approaches. **Results**: Pharmacological modulation of GPCR signaling produced non-linear and context-dependent effects. Compounds predicted to further increase excitability may instead promote phenotypic improvement, consistent with compensatory network rebalancing. Structural analyses revealed substantial divergence in ligand-binding pocket conservation for several GPCR-ligand pairs, suggesting that altered binding affinity and selectivity may also contribute to the observed phenotypic outcome. Pharmacological experiments performed in GPCR-depleted mutants allowed for the correlation of structural findings with functional effects for selected receptor-ligand pairs. Finally, genetic reduction in GPCRs coupled to stimulatory G proteins ameliorated hyperactive locomotion in *goa-1* mutants, whereas reduction in GPCRs coupled to inhibitory G proteins is largely insufficient to induce or exacerbate locomotor defects. **Conclusions**: Our findings identify excessive excitatory GPCR input as a key modulator of motor dysfunction in the context of impaired Gαo signaling. They also show that structural conservation is a necessary but not sufficient condition to predict functional responses. Overall, this study establishes *C. elegans* as a suitable platform to dissect GPCR-mediated signaling and highlights the value of integrating pharmacological and genetic approaches to guide target selection in *GNAO1*-related disorders.

## 1. Introduction

G protein-coupled receptors (GPCRs) mediate communication between neurons to regulate complex behaviors [1]. GPCRs transduce their signals by activating heterotrimeric G proteins, thereby promoting GTP binding to a Gα subunit and concomitant release of the Gβγ dimer. Dissociated Gα and Gβγ engage specific effectors to propagate signal. In striatal medium spiny neurons, Gαo, encoded by *GNAO1*, plays a key role in motor control, by transducing inhibitory signals from several GPCRs, including dopamine D2, adenosine A1, and cannabinoid receptors [2]. Gαo contributes to the regulation of intracellular cAMP levels by modulating the responsiveness of adenylyl cyclase 5 to stimulatory Gαs/Golf inputs through the release of Gβγ subunits [3]. In 2013, Nakamura and colleagues first identified dominant *GNAO1* variants in infantile neurological disorders characterized by severe movement disorders, with or without neurodevelopmental delay and epilepsy [4]. Susceptibility to a wide range of triggers, including emotion, fever, high environmental temperature, infections, intentional movements, and the sleep/wake cycle leads to severe exacerbation of movement disorders that represent the clinical signature of this condition [5]. *GNAO1*-associated dyskinetic episodes are life-threatening events that remain largely refractory to the current pharmacological treatments [6]. Benzodiazepines are used for acute management but require repeated high-dose administration, raising concerns about long-term safety [7]. At present, deep brain stimulation of the globus pallidus internus (GPi-DBS), and in some cases of the subthalamic nucleus (STN-DBS), represents the only intervention capable of providing sustained control of these episodes, despite its invasive nature [8,9].

Recent data from our group and others established that pathogenic variants in *goa-1*, ortholog of *GNAO1*, disrupt inhibitory Gαo signaling in *Caenorhabditis elegans* (*C. elegans*), leading to increased sensitivity to aldicarb (an acetylcholinesterase inhibitor), hyperactive locomotion, and accelerated egg-laying [10,11,12], phenocopying loss-of-function effects [13,14]. An increased frequency of backward movements (“reversals”) was documented for genetically modified *goa-1*[S47G], *goa-1*[A221D], and *goa-1*[E246K] strains, whereas worms carrying the R209H substitution crawled faster than controls with no major effects on the reversal rate. A milder increase in forward locomotion speed was also documented in *goa-1*[S47G] and *goa-1*[A221D] animals. In addition, pathogenic variants were shown to act in a cell-context-specific dominant-negative manner, where expression in specific classes of neurons antagonizes endogenous GOA-1 function, exacerbating behavioral defects. These findings provided a tractable *in vivo* platform for evaluating therapeutic interventions, and a pilot pharmacological screening identified caffeine, a non-selective adenosine receptor antagonist, as a compound able to improve aberrant locomotion in *goa-1* knock-in strains [10,12]. Caffeine significantly improved uncoordinated and hyperactive locomotion by blocking a putative adenosine receptor in the worm. While these findings provided the first evidence that *in vivo* modulation of GPCR signaling can ameliorate *GNAO1*-associated phenotypes, caffeine represents a compound whose pharmacology and clinical efficacy, although promising, remain to be fully elucidated. This raised the question of whether targeting additional GPCRs involved in motor control could provide alternative or complementary therapeutic strategies.

In striatal neurons, pathogenic *GNAO1* variants disrupt the integration of neuromodulatory signals mediated by adenosine and dopamine receptors, which act within the same neuronal populations to fine-tune motor output through Gαo-dependent pathways [3]. The adenosine-dopamine axis is evolutionarily conserved across species, including *C. elegans*, where adenosine signaling modulates dopaminergic function and locomotion [15,16]. Mechanistically, this modulation relies on antagonistic GPCR interactions that regulate neuronal excitability, analogous to those described in mammalian striatal circuits [17]. These findings suggest that the crosstalk between adenosine and dopamine GPCRs, crucial for motor regulation in mammals, is functionally conserved in the nematode. Importantly, disruption of inhibitory Gαo signaling, as observed in *GNAO1*-related disorders and in *goa-1* mutant worms, is predicted to unbalance this network toward excessive neuronal activation. This suggests that pharmacological inhibition of GPCRs coupled to stimulatory G proteins (Gs/Golf), or activation of GPCRs coupled to inhibitory G proteins (Gi), may partially restore signaling balance and ameliorate motor phenotypes. On this basis, dopamine D1-like receptors, which are coupled to stimulatory G proteins, and D2-like receptors, which signal through inhibitory G proteins, represent primary candidates for therapeutic modulation [18]. In addition, cannabinoid receptor signaling, which converges on Gi-coupled pathways and plays a well-established role in motor control, emerges as a rational complementary target [19].

Together, these considerations point to *C. elegans* as a tractable model to interrogate conserved GPCR signaling mechanisms and highlight the need for an integrated experimental strategy combining pharmacological and genetic approaches to dissect GPCR contributions to Gαo-dependent motor phenotypes, and to evaluate the feasibility and intrinsic limitations of targeting dopaminergic and cannabinoid receptors in *GNAO1*-related disorders.

## 2. Materials and Methods

*C. elegans* strains and maintenance. Culture conditions, maintenance and genetic crosses were carried out using standard techniques [20]. The Bristol N2 (wild-type animals), DG1856 *goa-1*(*sa734*), NL2099 *rrf-3*(*pk1426*), COP1863 *goa-1*(*knu751*), LX645 *dop-1*(*vs100*), LX702 *dop-2(vs105)*, DA1814 *ser-1*(*ok345*), and DA2250 *mgl-2*(*tm355*);*mgl-1*(*tm1811*) strains were provided by the *Caenorhabditis* Genetics Center (CGC, University of Minnesota, Minneapolis, MN, USA). The *mgl-2*(*tm355*) null strain was isolated from DA2250 by backcrossing to N2 animals. The *goa-1*[S47G], *goa-1*[R209H], and *goa-1*[E246K] knock-in strains were previously generated by CRISPR-Cas9 [10,12].

Targeted pharmacological screen. The response to agonists and antagonists selectively targeting human dopamine or cannabinoid receptors (Tocris, Bio-Techne SRL, Milan, Italy) was assessed as previously described [10]. Drugs were freshly dissolved in DMSO (SKF-81297 hydrobromide and LE-300; final DMSO concentration of 0.07%), ethanol (ACEA, AM4113, and L-741,626; final ethanol concentration of 0.2%), or water (sumanirole maleate). DMSO at this concentration was previously shown to have a negligible effect on locomotion in *goa-1* mutants and to markedly decrease reversal rate in N2 animals [10]. Conversely, 0.2% ethanol significantly affects forward locomotion, but not reversal frequency, in *goa-1*[R209H] animals and has a mild effect on reversal rate in N2. For this reason, all measurements were performed in parallel in worms treated with either the drug or the corresponding solvent control. Drug concentrations were selected based on our previous work on adenosine receptor ligands in *C. elegans* [10], in which GPCR-targeting compounds required approximately 10-to-20-fold higher concentrations than those typically used in *in vitro* systems to achieve measurable behavioral effects. This difference likely reflects the need for compounds to penetrate the cuticle and reach neuronal targets, and is influenced by factors such as uptake, efflux, and metabolism. Notably, this observation is compound- and context-dependent and cannot be generalized. Therefore, concentrations were chosen empirically, taking into account both our previous findings and *in vitro* activity ranges for each compound [21,22,23,24,25,26]. Based on these considerations, a concentration of 10 µM was used for most compounds, whereas L-741,626 was tested at 100 µM. Drugs were added to the agar plates containing 5 mM potassium phosphate buffer pH 6.0, 1 mM CaCl_2_, and 1 mM MgSO_4_. Plates were freshly prepared and seeded with 12.5 μL of *E. coli* OP50 bacterial culture. Locomotion speed and the number of reversals per minute were measured in L3 animals on the assay plate after 2 h of exposure, by using an automated tracking system. Reversals are defined as backward movements equal to at least one-fifth of the animal’s length, corresponding to the length of the pharynx. This protocol was recently validated for compounds targeting the adenosine receptors [10].

RNAi experiments. RNA interference (RNAi) was performed by feeding, as previously described [27], with minor modifications. Briefly, plates containing NGM agar, 1 mM IPTG and 50 μg/mL ampicillin were seeded with *E. coli* HT115 bacteria from the Ahringer *C. elegans* RNAi feeding library, and grown overnight at 37 °C. The day after, L3 hermaphrodites were transferred onto RNAi plates and incubated for 72 h at 15 °C. Adults were then moved to fresh RNAi plates and were allowed to lay eggs for 24 h at 20 °C before removal. Progeny were incubated at 20 °C until they reached the L3 stage to score for locomotion. As a negative control, RNAi plates were seeded with HT115 bacteria expressing the empty vector (L4440; EV). As a control for RNAi efficiency, *ama-1*(*RNAi*) was carried out and embryonic lethality was quantified. RNAi efficiency and specificity were also assessed by semi-quantitative RT-PCR analysis of target gene expression (Figure S1). Total RNA was extracted from adult worms treated with EV or gene-specific RNAi, and cDNA was synthesized using a SuperScript kit (Invitrogen, Carlsbad, CA, USA) with random oligonucleotides. Gene expression levels of *dop-1* and *dop-2* were analyzed using gene-specific primers (Table S1).

Construction of GPCR-ligand complexes and computation of the diversity score. When available, experimentally resolved human GPCR-ligand complexes were retrieved from the Protein Data Bank (PDB), including adenosine A2A receptor (A2AR)/caffeine (PDB ID: 3RFM [28]), A2AR/istradefylline (PDB ID: 8GNG [29]), and D1R/SKF-81297 hydrobromide (PDB ID: 7JV5 [30]). For all other GPCR-ligand pairs, receptor-ligand interactions were reconstructed by molecular docking using AutoDock Vina v.1.2 software package (https://vina.scripps.edu/, accessed on 12 May 2026) [31,32]. Receptor structures were reoriented prior to docking using the bio3d package (v.2.4-5) in R (https://thegrantlab.org/bio3d/, accessed on 12 May 2026), and docking was restricted to the central transmembrane region of the receptor, where GPCR ligand binding predominantly occurs [33,34,35]. The docking pose with the best predicted affinity score was selected for subsequent analyses. Ligand-binding sites were defined as all receptor residues containing at least one atom within 5 Å of any ligand atom, a threshold commonly used to identify GPCR binding pockets [36]. Conservation of binding-site residues between human GPCRs and their *C. elegans* orthologs was evaluated by accounting for amino acid substitutions and their physicochemical impact. Using this approach, we generated a structured dataset for each GPCR/ligand pair, analogous to that illustrated in Table S2 showing the remarkable conservation of the A2AR/caffeine interaction. It should be noted, however, that ADOR-1, the sole adenosine receptor identified in *C. elegans*, is annotated as an ortholog of multiple mammalian adenosine receptors, including A1R, A2AR, and A2BR (WormBase, WS298 release, 27 November 2025; https://www.wormbase.org/), and may therefore partially recapitulate functions attributed to different adenosine receptor subtypes in mammals.

For each GPCR-ligand pair, we computed a “diversity score” (*D_s_*), a quantitative descriptor integrating the magnitude of physicochemical changes at each binding-site position with the spatial proximity of the residue to the ligand. Residues closer to the ligand were assigned greater weight, reflecting their higher potential impact on ligand recognition. The *D_s_* was calculated as a distance-weighted average of the global disruption index across all binding-site residues:

$$D_{s}=\;\frac{\;\sum_{i=1}^{N_{res}}\left(\frac{G_{i}}{D_{i}}\right)}{\sum_{i=1}^{N_{res}}\left(\frac{1}{D_{i}}\right)}$$

where *G_i_* represents the global physicochemical disruption associated with the substitution at residue *i*, and *D_i_* denotes the spatial distance between residue *i* and the ligand in the human GPCR-ligand complex. *D_s_* values range from zero (0% divergence; complete conservation) to one (100% divergence, including gap substitutions). Details on the construction and validation of physicochemical substitution matrices are provided in the Supplementary Methods and Figures S2 and S3.

Statistics. Statistical analyses were performed using GraphPad Prism v.8.4.2 (https://www.graphpad.com/). Details on statistical tests, sample sizes, and data representation are provided in the figure legends and in Supplementary Tables. Sample size was determined based on previous studies using *goa-1* mutant strains [10,12]. No formal *a priori* power calculation was performed. Each experiment was conducted in at least three independent biological replicates, with the number of animals per condition reported in Tables S3–S8. To minimize potential confounders, all experimental groups were processed in parallel under identical environmental conditions. Drug-treated and solvent-treated controls were assayed simultaneously. Locomotor measurements were performed using an automated tracking system under standardized settings [10,12]. Genotype blinding was applied during behavioral assessment and data analysis.

## 3. Results

### 3.1. Targeted Pharmacological Screen of GPCR Ligands in C. elegans goa-1 Mutants

**Figure 1:**
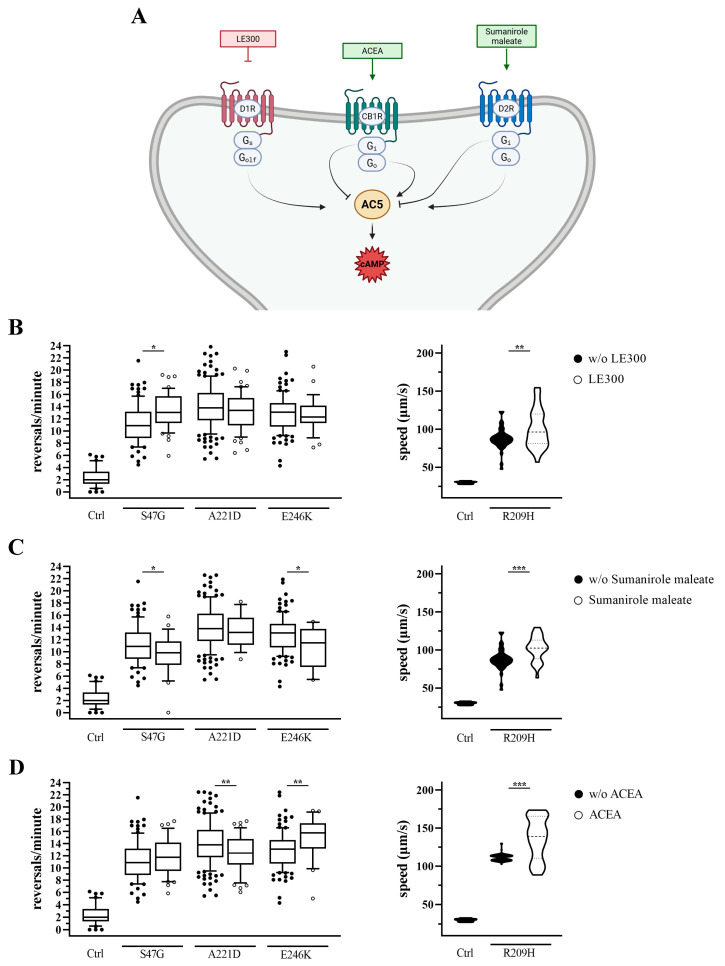
Effects of GPCR ligands predicted to ameliorate hyperactive locomotion in *goa-1* mutants. (**A**) Schematic overview of the GPCR signaling pathways and pharmacological agents investigated. In mammalian systems, dopamine D1-like receptors (D1R), coupled to stimulatory G proteins (Gs/Golf), and dopamine D2-like receptors (D2R), coupled to inhibitory G proteins (Gi/Go), regulate intracellular cAMP levels via adenylyl cyclase 5 (AC5). Cannabinoid type 1 receptors (CB1R), coupled to inhibitory G proteins, were included as additional modulators of Gαo signaling. These GPCR-G protein relationships are conserved in *C. elegans*. Green boxes indicate agonists; red boxes indicate antagonists. Created with BioRender.com. (**B**–**D**) Locomotor responses to GPCR-targeting compounds after 2 h exposure to (**B**) LE-300 (D1R antagonist), (**C**) sumanirole maleate (D2R agonist), and (**D**) ACEA (CB1R agonist). Reversal rate was measured in *goa-1*[S47G], *goa-1*[A221D], and *goa-1*[E246K] animals, while forward locomotion speed was assessed in *goa-1*[R209H] mutants. Data are expressed as mean ± SEM of at least three independent assays and normalized to solvent-treated controls. Statistical analysis was performed using an unpaired *t*-test with Welch’s correction (* *p* < 0.05; ** *p* < 0.01; *** *p* < 0.0005). Under untreated conditions, all *goa-1* mutant strains differed significantly from N2 (Ctrl) animals (*p* < 0.0001). The number of animals tested is reported in Tables S4 and S5.

**Figure 2:**
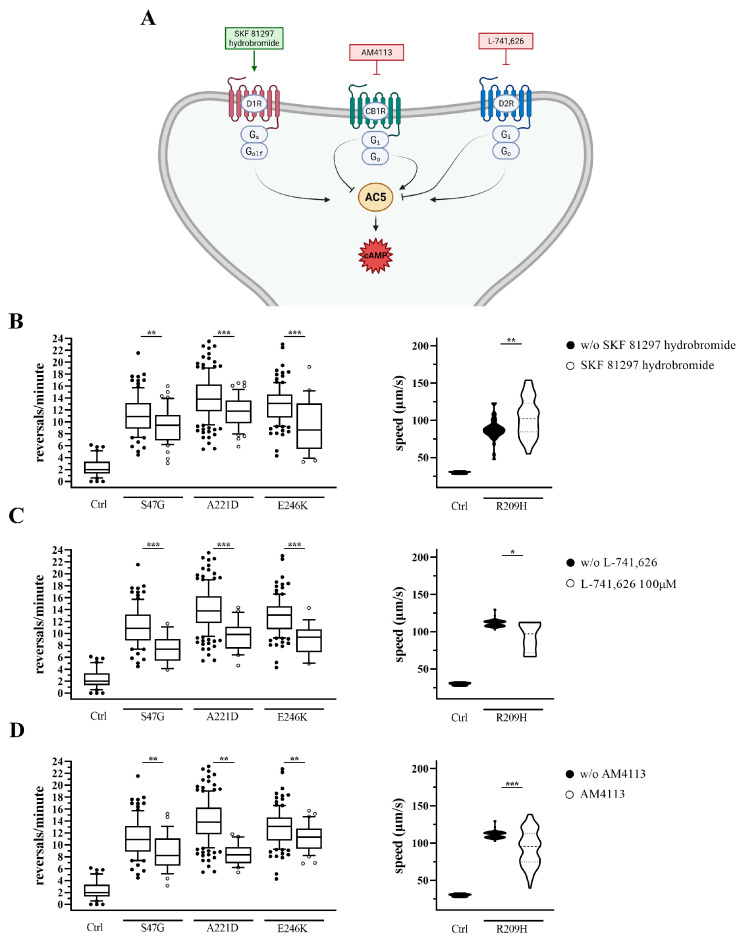
Effects of GPCR ligands predicted to exacerbate hyperactive locomotion in *goa-1* mutants. (**A**) Schematic overview of the GPCR signaling pathways and pharmacological agents investigated (see Figure 1 legend for details). Created with BioRender.com. (**B**–**D**) Locomotor responses to GPCR-targeting compounds after 2 h exposure to (**B**) SKF-81297 hydrobromide (D1R agonist), (**C**) L-741,626 (D2R antagonist), and (**D**) AM4113 (CB1R antagonist). Reversal rate was measured in *goa-1*[S47G]*, goa-1*[A221D]*,* and *goa-1*[E246K] animals, while forward locomotion speed was assessed in *goa-1*[R209H] mutants. Data are expressed as mean ± SEM of at least three independent assays and normalized to solvent-treated controls. Statistical analysis was performed using an unpaired *t*-test with Welch’s correction (* *p* < 0.05; ** *p* < 0.01; *** *p* < 0.0005). Under untreated conditions, all *goa-1* mutant strains differed significantly from N2 (Ctrl) animals (*p* < 0.0001). The number of animals tested is reported in Tables S4 and S5.

Building on our previous findings indicating that adenosine receptor antagonists (e.g., caffeine and istradefylline) ameliorate aberrant locomotion in *goa-1* mutants [10,12], we performed a targeted pharmacological screening of GPCR ligands to evaluate their effects on *C. elegans* locomotion (Figure 1A and Figure 2A). The strategy was based on the premise that, in a genetic background characterized by impaired inhibitory Gαo signaling, pharmacological inhibition of GPCRs coupled to stimulatory G proteins (Gs/Golf), or activation of GPCRs coupled to inhibitory G proteins (Gi), are predicted to attenuate excessive neuronal excitability, thus ameliorating the phenotype, whereas the opposite interventions are expected to exacerbate motor defects.

We first evaluated the effect of a selective agonist and a selective antagonist of the human dopamine D1 receptor (D1R; SKF-81297 hydrobromide and LE-300, respectively) [37,38], which act upstream of stimulatory G proteins, promoting adenylyl cyclase activity and cAMP production in mammalian systems [39]. We then analyzed the effect of selective agonists/antagonists of the dopamine D2 receptor (D2R; sumanirole maleate and L-741,626, respectively) [40,41], and of the cannabinoid receptor type 1 (CB1R; ACEA and AM4113, respectively) [21,42], both of which act upstream of inhibitory G proteins [39,43]. Following 2 h of drug exposure, the effect was evaluated in terms of reversal rate (*goa-1*[S47G], *goa-1*[A221D], and *goa-1*[E246K]) and forward locomotion speed (*goa-1*[R209H]). The choice of these parameters reflects the distinct locomotor phenotypes associated with each *goa-1* variant [10,12] and allows for a sensitive assessment of drug effects in diverse genetic backgrounds. As a positive control, we assessed the effect of caffeine in a selected panel of *goa-1* mutants. Consistent with our previous findings [10,12], caffeine significantly ameliorated hyperactive locomotion, as measured by reversal rate (Figure S4), and was systematically used in *goa-1*[S47G] animals as an internal control in each experiment. Drug treatments were also performed in N2 animals (Table S3). Among the compounds tested, only the D1R antagonist LE-300 produced a statistically significant effect, increasing the reversal rate compared to solvent-treated controls.

Among the compounds predicted to ameliorate hyperactive locomotion in *goa-1* knock-in strains (Figure 1; Tables S4 and S5), LE-300 (a D1R antagonist) did not improve locomotion in any mutant; instead, it exacerbated locomotion speed in *goa-1*[R209H] and reversal rate in *goa-1*[S47G] animals (Figure 1B). In contrast, sumanirole maleate (a D2R agonist) slightly reduced the reversal rate of *goa-1*[S47G] and *goa-1*[E246K] animals, but negatively affected locomotion speed (Figure 1C). Finally, ACEA (a CB1R agonist) worsened locomotion speed and mildly affected the reversal rate, with opposite effects observed in *goa-1*[A221D] and *goa-1*[E246K] animals (Figure 1D).

Unexpectedly, compounds predicted to exacerbate hyperactive locomotion instead exhibited marked beneficial effects across all mutant strains, with the exception of SKF-81297 hydrobromide (a D1R agonist), which improved reversal rate but not locomotion speed (Figure 2B–D; Tables S4 and S5).

These findings indicate that pharmacological modulation of GPCR signaling in the context of impaired Gαo function may yield non-linear and context-dependent outcomes. In a neuromodulatory network disrupted by *goa-1* mutations, compounds predicted to further increase excitability may instead promote compensatory rebalancing of circuit activity, possibly through receptors crosstalk/heterodimerization, or coupling to alternative signaling pathways. In addition, selective ligands developed for human GPCRs may display altered pharmacological profiles across species, a phenomenon widely documented for multiple receptor families [44]. In line with that, minor sequence variations within ligand-binding pockets can lead to substantial differences in ligand affinity, efficacy, or functional selectivity, including switches between agonism and antagonism. These considerations prompted us to investigate whether differences in ligand-binding pocket conservation between human and nematode GPCRs could account, at least in part, for the observed phenotypic outcomes.

### 3.2. Structural Conservation of GPCR Ligand-Binding Sites and Associated Functional Responses

Drawing on established GPCR-ligand interactions in mammals, we implemented a computational framework to evaluate the structural and physicochemical conservation of ligand-binding pockets between human GPCRs and their *C. elegans* orthologs. The GPCR/ligand interfaces analyzed in this study are reported in Table 1. For each GPCR-ligand pair, we quantified the degree of conservation by defining a “diversity score” (*D_s_*), a numerical descriptor that integrates amino acid substitutions within the binding pocket with their physicochemical impact and spatial proximity to the ligand. By definition, higher *D_s_* values indicate greater divergence of the binding site, whereas lower values reflect stronger structural conservation between species. Structural data were obtained from experimentally determined GPCR-ligand complexes available in the Protein Data Bank or, when unavailable, from molecular docking reconstructions. Binding-site residues were identified based on spatial proximity to the ligand, and amino acid substitutions between human and nematode receptors were evaluated according to key physicochemical properties (i.e., size, hydrophobicity, polarity, and charge). These features were combined into a global disruption index, which was then weighted by the distance of each residue from the ligand to compute the *D_s_* value (see Section 2 and Supplementary Methods for details).

**Figure 3:**
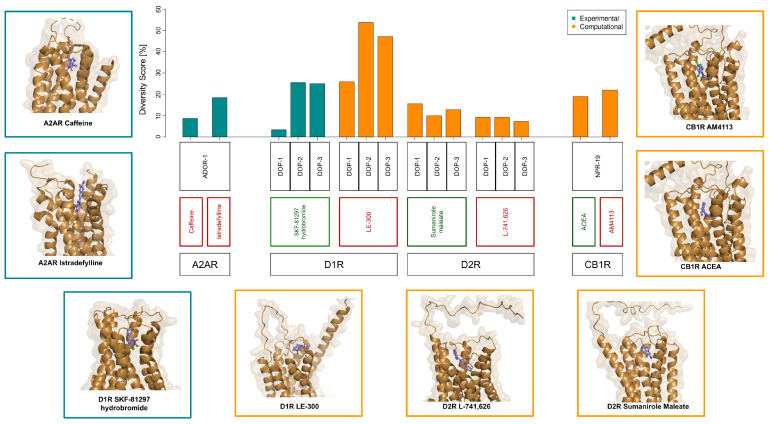
Diversity score of GPCR ligand-binding sites between human and *C. elegans*. The diversity score (*D_s_*) was calculated for each GPCR/ligand pair to quantify the degree of structural and physicochemical divergence between human receptors and their *C. elegans* orthologs. For each entry, the bottom label indicates the human GPCR, the central label indicates the corresponding ligand (green and red denote agonists and antagonists, respectively), and the top label indicates the *C. elegans* ortholog. Bars represent *D_s_* values and are colored according to the source of the GPCR/ligand complex used for the analysis: green bars indicate experimentally determined structures retrieved from the Protein Data Bank, whereas orange bars indicate complexes reconstructed by molecular docking. Lower *D_s_* values correspond to higher conservation of the ligand-binding site. Representative molecular images are shown alongside the bar plot to illustrate ligand positioning within the binding pocket for both experimentally determined and docking-derived complexes, facilitating a qualitative assessment of docking results.

Using this approach, we observed a relatively high degree of conservation for the caffeine/ADOR-1 interaction (*D_s_* < 10%) (Figure 3), consistent with previous evidence supporting a preserved caffeine-adenosine receptor pathway in *C. elegans* [15,16,45]. In contrast, lower conservation was observed for the interaction between ADOR-1 and the selective A2AR antagonist istradefylline, and between CB1R ligands and the *C. elegans* cannabinoid receptor NPR-19 (*D_s_* ~20%). Among dopamine receptor ligands, the binding site for the D1R agonist SKF-81297 hydrobromide showed high conservation in the nematode D1-like receptor DOP-1, but not in the D2-like receptors DOP-2 and DOP-3, suggesting high specificity for DOP-1. In contrast, the binding site for the D1R antagonist LE-300 displayed low conservation across all dopamine receptor subtypes in *C. elegans*. Finally, binding sites for both D2R ligands showed comparable levels of conservation across multiple dopamine receptors, consistent with limited selectivity.

To directly assess the relationship between structural predictions and functional outcomes, we performed pharmacological experiments in receptor-depleted backgrounds using *goa-1*[S47G];*dop-1*(0) and *goa-1*[S47G];*dop-2*(0) double mutant strains.

**Figure 4:**
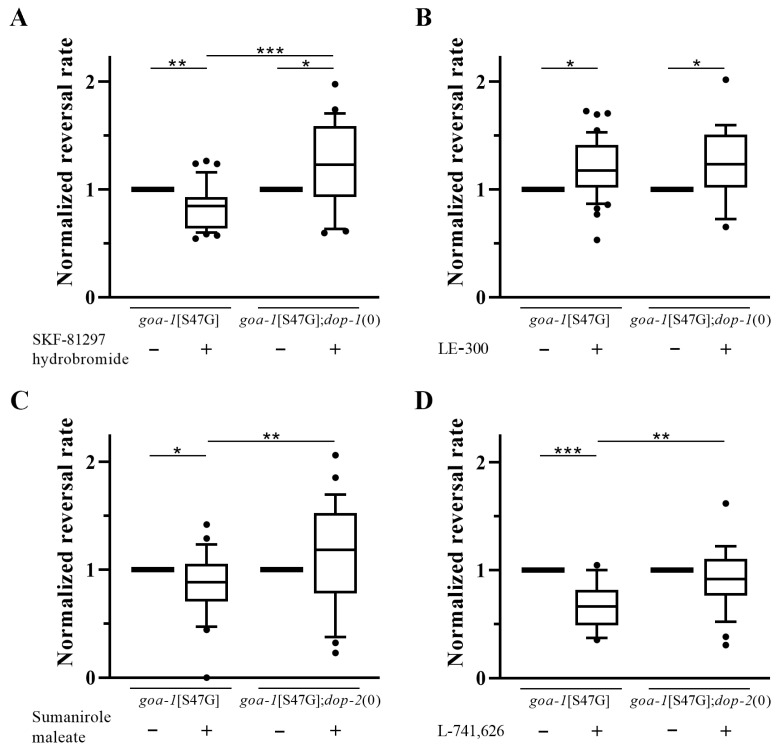
Functional validation of D1R and D2R ligand effects in receptor-depleted backgrounds. (**A**) SKF-81297 hydrobromide (D1R agonist) improved the hyperactive reversal phenotype in *goa-1*[S47G] animals, but this effect was abolished in the *dop-1*(0) background. (**B**) LE-300 (D1R antagonist) slightly worsened the phenotype, with no significant change in *dop-1*(0) animals. (**C**) Sumanirole maleate (D2R agonist) produced a mild improvement that was abolished in the *dop-2*(0) background. (**D**) L-741,626 (D2R antagonist) markedly improved the phenotype, and this effect was almost abolished in *dop-2*(0) animals. These results are consistent with structural predictions and suggest receptor-specific contributions to the observed effects for specific GPCR-ligand pairs. Statistical significance was assessed using an unpaired *t*-test with Welch’s correction (* *p* < 0.05; ** *p* < 0.01; *** *p* < 0.001). Data are normalized to the corresponding solvent-treated control. Sample size is reported in Table S4.

Structural analysis predicted a high degree of binding-site conservation between the D1R agonist SKF-81297 hydrobromide and the DOP-1 receptor (*D_s_* = 3.3%), with substantially lower conservation for DOP-2 and DOP-3 (*D_s_* ≅ 25.0%). Consistent with this, the phenotypic improvement observed in *goa-1*[S47G] animals treated with this compound was abolished in the *dop-1*(0) background, where treatment resulted in a significant worsening of the reversal rate compared to solvent-treated controls (Figure 4A; Table S4). These findings indicate that the effect of SKF-81297 hydrobromide is largely mediated by DOP-1. However, the direction of the phenotypic response cannot be explained by canonical receptor coupling alone, suggesting the involvement of context-dependent network effects or altered functional activity of the ligand at the nematode receptor. In contrast, the D1R antagonist LE-300 displayed low binding-site conservation for DOP-1 (*D_s_* = 25.9%) and poor conservation for DOP-2 and DOP-3 (*D_s_* = 53.8 and 47.1, respectively). In line with this, the mild worsening of the phenotype observed in *goa-1*[S47G] animals treated with LE-300 did not change in the *dop-1*(0) background (Figure 4B; Table S4), indicating that this effect is unlikely to be mediated by DOP-1. This observation suggests that LE-300 may act through alternative GPCR targets or via GPCR-independent mechanisms.

Regarding the D2R ligands, the agonist sumanirole maleate displayed a relatively high degree of binding-site conservation for DOP-2 (*D_s_* = 10.0%) with moderately lower conservation for DOP-1 (*D_s_* = 15.5%). Consistent with this prediction, the mild improvement of the phenotype observed in *goa-1*[S47G] animals treated with this compound was abolished in the *dop-2(0)* background (Figure 4C; Table S4), indicating that the effect of sumanirole maleate is primarily mediated by DOP-2 and supporting the predictive value of the structural analysis for this ligand-receptor pair. Finally, the D2R antagonist L-741,626 showed high but comparable levels of binding-site conservation across the three dopamine receptors, suggesting limited selectivity. In *goa-1*[S47G] animals, L-741,626 markedly improved the hyperactive reversal phenotype, but this effect was almost abolished in the *dop-2(0)* background (Figure 4D; Table S4), indicating that DOP-2 is required for the observed phenotypic response. Despite the predicted binding-site conservation in additional dopamine receptors, these results suggest that DOP-2 plays a dominant role in mediating the effect of L-741,626 in this context, although contributions from other receptors or compensatory interactions cannot be ruled out.

Together, these findings show that structural predictions may help identify receptor-specific contributions to the observed pharmacological effects on the phenotype. It is important to note, however, that binding-site conservation should be interpreted as a necessary but not sufficient condition for functional conservation, as it does not predict the nature of receptor activation and the direction of functional outcomes. To complement pharmacological approaches, we therefore employed genetic strategies based on RNAi-mediated knockdown or receptor knockout to directly assess GPCR contributions to Gαo-dependent motor phenotypes.

### 3.3. Genetic Dissection of GPCR Signaling Pathways Modulating Hyperactive Locomotion in goa-1 Mutants

**Figure 5:**
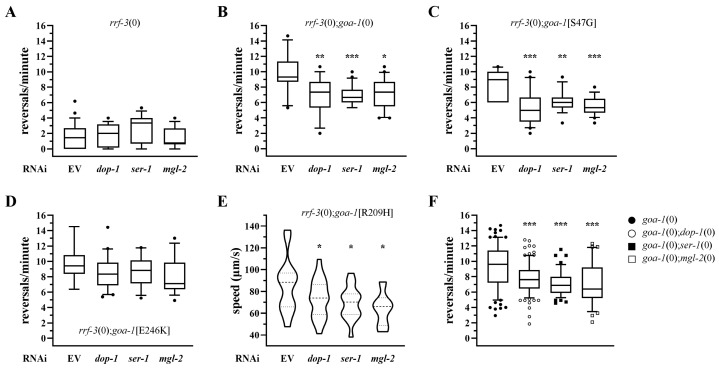
Genetic reduction in GPCRs coupled to stimulatory G proteins ameliorates hyperactive locomotion in *goa-1* mutants. RNAi-mediated silencing of GPCRs coupled to stimulatory G proteins was performed in *goa-1* mutant backgrounds using the RNAi-hypersensitive *rrf-3(pk1426)* null (0) strain. (**A**) Locomotor behavior of *rrf-3*(0) animals treated with the empty vector (EV) or RNAi targeting *dop-1*, *ser-1*, or *mgl-2*. (**B**–**E**) Reversal rate measured in *goa-1*(0), *goa-1*[S47G], *goa-1*[R209H], and *goa-1*[E246K] animals upon RNAi-mediated knockdown of the target genes. (**F**) Validation of RNAi results using a genetic loss-of-function approach. The reversal rate was measured in *goa-1*(0) animals crossed with *dop-1*, *ser-1*, or *mgl-2* knockout strains. Data are expressed as mean ± SEM of at least three independent assays and are normalized to EV-treated controls. Statistical analysis was performed using an unpaired *t*-test with Welch’s correction (* *p* < 0.05; ** *p* < 0.01; *** *p* < 0.005). The number of animals tested are reported in Tables S6–S8.

Genetic approaches were used to reduce/abolish GPCR signaling in a receptor-specific and ligand-independent manner. We first silenced, by RNAi, genes encoding GPCRs coupled to stimulatory G proteins in *goa-1* mutant backgrounds, including *dop-1*, *ser-1* (a serotonergic receptor), and *mgl-2* (a metabotropic glutamate receptor). These GPCRs were selected as representatives of distinct neuromodulatory systems converging on excitatory signaling pathways [46,47,48]. Experiments were performed in the RNAi-hypersensitive *rrf-3(pk1426)* null (0) background, which allows efficient neuronal silencing, and included three *goa-1* knock-in mutants and the *goa-1*(*sa734*) null (0) strain. RNAi-mediated knockdown of *dop-1*, *ser-1*, and *mgl-2* significantly improved the hyperactive locomotor phenotype observed in *goa-1*[S47G] and *goa-1*[R209H] animals, as well as in the *goa-1*(0) strain (Figure 5B,C,E; Tables S6 and S7), without affecting locomotion in *rrf-3*(0) worms (Figure 5A; Table S6). Only a mild, non-significant effect on reversal rate was observed in *goa-1*[E246K] animals (Figure 5D; Table S6).

We next validated these results using a genetic loss-of-function approach by analyzing locomotion in *goa-1*(0) animals crossed with *dop-1*, *ser-1*, or *mgl-2* null strains (Figure 5F; Table S8). We focused on the *goa-1*(0) background since it provides a robust and genetically homogeneous phenotype independent of allele-specific effects. In all cases, the phenotypic rescue observed in the double mutants closely mirrored the effects obtained by RNAi, supporting the conclusion that reduced activity of stimulatory GPCRs is sufficient to ameliorate hyperactive locomotion in the context of impaired Gαo signaling.

**Figure 6:**
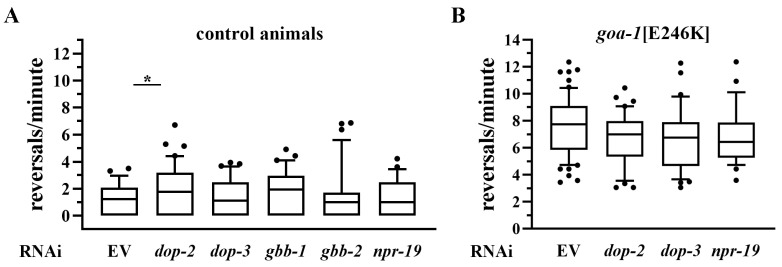
Genetic reduction in GPCRs coupled to inhibitory G proteins has limited effects on locomotion. RNAi-mediated knockdown of GPCRs acting upstream of inhibitory Gi/o proteins was performed in *rrf-3*(0) animals (**A**) and in *goa-1*[E246K] mutants (**B**). Reversal rate was measured following silencing of *dop-2*/*dop-3* (D2-like dopamine receptors), *gbb-1*/*gbb-2* (GABAB receptor subunits), or *npr-19* (cannabinoid receptor). RNAi treatment produced only minor effects on locomotion, with a modest increase in reversal rate observed upon *dop-2* silencing. Data are expressed as mean ± SEM of at least three independent assays and are normalized to EV-treated controls. Statistical analysis was performed using an unpaired *t*-test with Welch’s correction (* *p* < 0.05). The number of animals tested are reported in Table S6.

To assess whether reduced inhibitory GPCR signaling is able to induce hyperactive locomotion recapitulating the phenotype observed in *goa-1* mutants, we silenced GPCRs acting upstream of inhibitory Gi/o proteins in *rrf-3(0)* animals. Specifically, we targeted *dop-2* and *dop-3*, encoding D2-like dopamine receptors [46], *gbb-1* and *gbb-2*, encoding the two subunits of the GABAB receptor [49], and *npr-19*, encoding a cannabinoid receptor [50]. Unlike the effects observed in *goa-1* mutants upon silencing of stimulatory GPCRs, RNAi-mediated knockdown of Gi/o-coupled receptors produced only minor effects in wild-type animals, with a mild increase in reversal rate observed only upon *dop-2* silencing (Figure 6A; Table S6).

To further assess the contribution of GPCRs coupled to inhibitory G proteins, we performed RNAi-mediated knockdown of *dop-2*, *dop-3*, and *npr-19* in *goa-1*[E246K] animals. No significant changes in reversal rate were observed compared to controls (Figure 6B; Table S6), indicating that additional reduction in inhibitory input does not exacerbate the phenotype under conditions of impaired Gαo signaling. We also investigated genetic interactions using double mutants. However, the interpretation of data from *goa-1*[S47G];*dop-2*(0) animals is not informative, as the *dop-2(vs105)* loss-of-function allele is associated with intrinsic locomotor defects, including altered backward movement [51], which corresponds to the reversal behavior used as the primary readout in this study (Figure S5; Table S4). Of note, *dop-1* knockout significantly ameliorated the phenotype of *goa-1*[S47G] animals (Figure S5; Table S4), consistent with the effects observed in Figure 4.

Together, these results indicate that hyperactive locomotion emerges primarily when inhibitory Gαo signaling is compromised, allowing excessive excitatory GPCR input to act as a key modulator of the phenotype. By contrast, genetic reduction in GPCRs coupled to inhibitory G proteins is largely insufficient to induce locomotor abnormalities *per se*, suggesting that disruption of individual inhibitory GPCR inputs is not sufficient to recapitulate the effects of global Gαo impairment.

## 4. Discussion

*GNAO1*-related disorders are rare but devastating neurodevelopmental conditions characterized by severe, often life-threatening paroxysmal movement disorders that frequently require intensive care admission [5,6]. Current therapeutic options remain limited. Benzodiazepines are widely used for acute management, but their efficacy relies on global suppression of neuronal activity and is associated with tolerance, respiratory depression, and significant long-term risks [7]. DBS implantation improves dystonia and reduces motor exacerbations with an acceptable safety profile [9], but represents an invasive intervention and is not universally effective. Together, these limitations highlight a critical unmet need for targeted, mechanism-based therapies aimed at preventing or attenuating paroxysmal dyskinetic episodes rather than globally suppressing neuronal activity.

Gαo is a central inhibitory signaling hub in striatal medium spiny neurons, where it integrates inputs from multiple GPCRs involved in motor control, including dopamine, adenosine, serotonin, metabotropic glutamate, and cannabinoid receptors [2]. Disruption of Gαo signaling by pathogenic *GNAO1* variants is therefore expected to impair the fine-tuning of excitation/inhibition balance within basal ganglia circuits [3]. In this context, GPCRs are particularly attractive therapeutic targets, as they are highly druggable, account for a substantial fraction of approved medications, and enable both inhibitory and stimulatory modulation of neuronal activity [1,52].

Our previous work established caffeine as a compound capable of robustly rescuing aberrant locomotion in *goa-1 C. elegans* models of *GNAO1*-related disorders [10,12]. Pharmacological experiments using selective adenosine receptor ligands indicated that part of this effect is mediated through antagonism of an adenosine receptor in the worm [10]. However, the reduced and genotype-dependent efficacy of the selective A2AR antagonist istradefylline suggested that caffeine acts through both adenosine receptor-dependent and -independent mechanisms [12]. This is consistent with the known pleiotropic pharmacology of caffeine, which at increasing concentrations inhibits phosphodiesterases, mobilizes intracellular calcium, and modulates multiple neuromodulatory pathways [53,54]. Notably, although anecdotal reports of caffeine use exist in *GNAO1* patients, and beneficial effects have been reported in related disorders such as *ADCY5*- and *PDE2A*-associated dyskinesia [55,56], its clinical efficacy in *GNAO1*-related disorders has not yet been formally evaluated in controlled studies. This highlights the importance of further investigating caffeine-based interventions while also identifying additional or complementary therapeutic targets.

To this end, given the central role of dopamine and cannabinoid signaling in the physiology and physiopathology of the basal ganglia [43], we selected agonists and antagonists of D1R, D2R, and CB1R for a targeted pharmacological screening. This choice was further motivated by clinical evidence showing that modulation of dopamine signaling with vesicular monoamine transporter 2 (VMAT2) inhibitors, such as tetrabenazine, is commonly used to manage baseline movement disorders in subjects with *GNAO1* pathogenic variants, albeit with limited efficacy for paroxysmal events [6]. Although we did not directly measure cAMP levels in *C. elegans*, the pharmacological profile of the compounds used in this study is well characterized in mammalian systems. D1-like receptor agonists, including SKF-81297 hydrobromide, are known to stimulate cAMP production via Gs/Golf activation [37,57,58], whereas D2-like receptor agonists, including sumanirole maleate, and CB1 receptor agonists, including ACEA, inhibit cAMP accumulation through Gi/o signaling [40,42,59]. Conversely, antagonists of the same receptors exert opposite effects on cAMP signaling [21,38,41,60]. These established properties provided the rationale for classifying compounds as predicted modulators of excitatory or inhibitory GPCR signaling in our experimental design. However, the extent to which these effects are conserved in *C. elegans* remains uncertain, particularly in light of the structural divergence of ligand-binding pockets and the intrinsic difficulty in predicting the functional outcome of ligand-receptor interactions across species. More broadly, cross-species differences in GPCR pharmacology represent an important limitation of this study. In *C. elegans*, ligand-binding pockets, receptor repertoires, and downstream signaling contexts may differ substantially from those of mammalian systems, thereby affecting both ligand selectivity and functional outcomes [44]. Although some GPCR-targeting compounds, such as caffeine and dopaminergic ligands, have been previously investigated in this organism [10,15,16], to our knowledge, among the ligands tested in this study, only the CB1R agonist ACEA has been previously applied in *C. elegans* in the context of food preference behavior [61]. These considerations highlight the need for caution when interpreting pharmacological data across species.

Consistent with that, compounds predicted to exacerbate hyperactive locomotion through inhibition of GPCRs coupled to inhibitory G proteins or activation of GPCRs coupled to stimulatory G proteins instead produced phenotypic improvement in *goa-1* mutants. This observation is consistent with the idea that *GNAO1* variants may perturb the neuromodulatory network, and that pharmacological interventions can trigger compensatory rebalancing rather than simple linear, receptor-specific effects. In support of this model, recent findings by Solis and colleagues [62] indicate that a significant number of *GNAO1* variants associated with severe clinical features disrupt the global neuronal GPCR signaling network through neomorphic interactions of mutant Gαo with RIC8 proteins, obligate chaperones of all Gα subunits, resulting in RIC8 mislocalization from the cytoplasm to the Golgi apparatus. At the same time, our structural analyses revealed substantial divergence in ligand-binding pocket conservation between human and *C. elegans* GPCRs. Together, these findings suggest that both network-level alterations and limited structural conservation contribute to the divergence between predicted and observed pharmacological effects. An alternative strategy to overcome limitations imposed by poor binding-site conservation is the generation of humanized *C. elegans* strains, in which the endogenous coding sequence is replaced by the human sequence, enabling pharmacological interrogation with human-selective ligands [63,64]. Using this approach, Larasati and colleagues recently described a humanized *Drosophila melanogaster* model carrying the recurrent G203R pathogenic *GNAO1* variant, in which zinc supplementation restored motor function and improved survival [65].

As a complementary approach, we employed a genetic strategy to interrogate GPCR function independently of ligand binding properties and cross-species variability. In *goa-1* mutant backgrounds, RNAi-mediated silencing and genetic knockout of GPCRs coupled to stimulatory G proteins consistently ameliorated hyperactive locomotion, whereas silencing of GPCRs coupled to inhibitory G proteins produced only minor effects. This asymmetry suggests that when Gαo signaling is compromised, excessive excitatory GPCR input becomes the dominant driver of motor dysfunction, whereas silencing single GPCRs regulating inhibitory pathways is not sufficient to induce pathology in a wild-type genetic background nor to exacerbate the phenotype in *goa-1* mutants. These findings indicate that reducing excitatory inputs may represent a more effective therapeutic strategy than further modulating inhibitory signaling in *GNAO1*-related disorders. Notably, caffeine produced a more robust rescue of the hyperactive phenotypes in *goa-1* mutants than either RNAi- or knockout-mediated silencing of individual excitatory GPCRs, an effect that likely reflects its multi-target nature. Unlike selective GPCR-targeting compounds, caffeine simultaneously modulates adenosine signaling, cyclic nucleotide metabolism, and neuronal excitability [53,54]. While this pleiotropy complicates mechanistic interpretation, it may also contribute to its higher efficacy. These observations raise the possibility that combinatorial strategies, combining caffeine with more selective modulators of excitatory GPCR pathways, could achieve greater therapeutic benefit with improved specificity. Finally, variability in treatment response across different *goa-1* alleles, including the reduced responsiveness of the E246K variant observed both in the present study and in previous pharmacological experiments [12], highlights the importance of allele-specific effects and supports a precision medicine approach to *GNAO1*-related disorders.

In conclusion, our study demonstrates that *C. elegans* provides a powerful platform to dissect conserved GPCR signaling networks relevant to Gαo signaling, while also highlighting the need for caution when translating pharmacological findings across species. By integrating pharmacological and genetic approaches, we identify excessive excitatory GPCR signaling as a key driver of hyperactive locomotion in the context of impaired Gαo function. These findings provide a conceptual framework for future therapeutic development and support the use of complementary strategies to guide target selection in rare movement disorders.

## Figures and Tables

**Table 1:** GPCR-ligand interactions analyzed in this study.

Compound	Receptor	Role	*C. elegans* Ortholog(s)
Caffeine	ARs	Antagonist	ADOR-1 (Uniprot ID: Q1ZXT1)
Istradefylline	A2AR (Uniprot ID: P29274)	Antagonist	ADOR-1 (Uniprot ID: Q1ZXT1)
SKF-81297 hydrobromide	D1R (Uniprot ID: P21728)	Agonist	DOP-1 (Uniprot ID: Q86ME6)
LE-300	D1R (Uniprot ID: P21728)	Antagonist	DOP-1 (Uniprot ID: Q86ME6)
Sumanirole maleate	D2R (Uniprot ID: P14416)	Agonist	DOP-2 (Uniprot ID: E7EM37) DOP-3 (Uniprot ID: Q6RYS9)
L-741,626	D2R (Uniprot ID: P14416)	Antagonist	DOP-2 (Uniprot ID: E7EM37) DOP-3 (Uniprot ID: Q6RYS9)
ACEA	CB1R (Uniprot ID: P21554)	Agonist	NPR-19 (Uniprot ID: Q17594)
AM4113	CB1R (Uniprot ID: P21554)	Antagonist	NPR-19 (Uniprot ID: Q17594)

A2AR, adenosine 2A subtype receptor; ARs, adenosine receptors; D1R, Dopamine D1 subtype receptor; D2R, Dopamine D2 subtype receptor; CB1R, Cannabinoid CB1 subtype receptor.

## Data Availability

The original contributions presented in this study are included in the article/Supplementary Material. Further inquiries can be directed to the corresponding author.

## Supplementary Materials

The following supporting information can be downloaded at: https://www.mdpi.com/article/10.3390/biomedicines14051139/s1. Supplementary Methods; Figure S1: Validation of RNAi efficiency and specificity by semi-quantitative RT-PCR; Figure S2: Substitution matrices; Figure S3: Comparison between substitution matrices; Figure S4: Caffeine suppresses the increased reversal rate in *goa-1* mutants; Figure S5: Effect of *dop-1* and *dop-2* loss-of-function on locomotion in *goa-1*[S47G] mutants; Table S1: Primer pairs and conditions used in RT-PCR assays; Table S2: Conservation of the AR/caffeine interaction between human and *C. elegans* GPCRs; Table S3: Reversal rate of wild-type (N2) animals exposed to selective agonists or antagonists targeting relevant GPCRs; Table S4: Reversal rate of *goa-1* mutants exposed to selective agonists or antagonists targeting relevant GPCRs; Table S5: Forward locomotion speed of the *goa-1*[R209H] mutant exposed to selective agonists or antagonists targeting relevant GPCRs; Table S6: Reversal rate in *C. elegans* strains following RNAi-mediated knockdown of genes encoding GPCRs acting upstream of inhibitory or stimulatory G proteins; Table S7: Forward locomotion speed measured in *rrf-3*(0);*goa-1*[R209H] animals following RNAi-mediated knockdown of genes encoding GCPRs acting upstream to stimulatory G proteins; Table S8: Reversal rate in *goa-1*(0) animals crossed with strains knockout for genes encoding GPCRs acting upstream to stimulatory G proteins. References [66,67,68,69,70] are cited in the supplementary materials.

## Abbreviations

The following abbreviations are used in this manuscript:

GPi-DBS	Deep brain stimulation of the globus pallidus internus
STN-DBS	Deep brain stimulation of the subthalamic nucleus
*C. elegans*	*Caenorhabditis elegans*
RNAi	RNA interference
GPCR	G protein-coupled receptor
D1R	Dopamine D1-like receptors
D2R	Dopamine D2-like receptors
CB1R	Cannabinoid type 1 receptors
AR	Adenosine receptor
A1R	Adenosine type 1 receptors
A2AR	Adenosine type 2A receptors
D_s_	Diversity score
VMAT2	Vesicular monoamine transporter 2
