# Supplementary material for: Dissecting GPCR Contributions to Gαo-Dependent Motor Dysfunction in GNAO1-Related Disorders Using Caenorhabditis elegans
Source: Biomedicines. 2026 May 18;14(5):1139. doi: 10.3390/biomedicines14051139 (PMC13204887; doi:10.3390/biomedicines14051139)
Supplement: Supplementary file 1 [file biomedicines-14-01139-s001.zip › biomedicines-4181150-supplementary.pdf]

Supplementary Materials and Methods

Supplementary Methods

Generation of substitution matrices. To evaluate the physicochemical impact of amino acid substitutions, we constructed custom substitution matrices based on established quantitative descriptor scales. Each scale assigns numerical values to amino acids with respect to a specific property: size, hydrophobicity, polarity, and charge [66-69]. The effect of a substitution was quantified as the absolute difference between the descriptor values of the original and substituted residue. To account for alignment gaps, each matrix was extended with an additional column corresponding to substitutions involving alignment gaps. In such cases, irrespective of the identity of the original residue, the maximum observed difference among all pairwise amino acid comparisons for the given descriptor was assigned. All values within each matrix were subsequently normalized by the maximum observed value for that descriptor, ensuring that substitution effects could be compared across properties with differing numerical ranges and units. The resulting normalized substitution matrices for each of the four descriptors are presented in Figure S2A. A composite substitution matrix was then generated by computing the arithmetic mean of the values across the four individual matrices (Figure S2B), which was used to represent the global physicochemical impact of amino acid substitutions. By construction, a value of zero denotes full residue conservation, while a value of one corresponds to substitutions involving gaps. To validate our substitution scores, we compared them with established substitution matrices, including BLOSUM62 and PAM120, which are derived from the observed amino acid replacement frequencies in homologous proteins. We also included comparisons with classical physicochemical-based substitution matrices, such as those proposed by Grantham et al. [68] and Miyata et al. [70]. Pearson correlation coefficients were computed between our custom matrices and those from the literature, with values reported in Figure S3. The relatively high correlations support the validity of our approach, while their moderate magnitude highlights the need for a dedicated matrix tailored to the specific objectives of this study.

Supplementary Figures

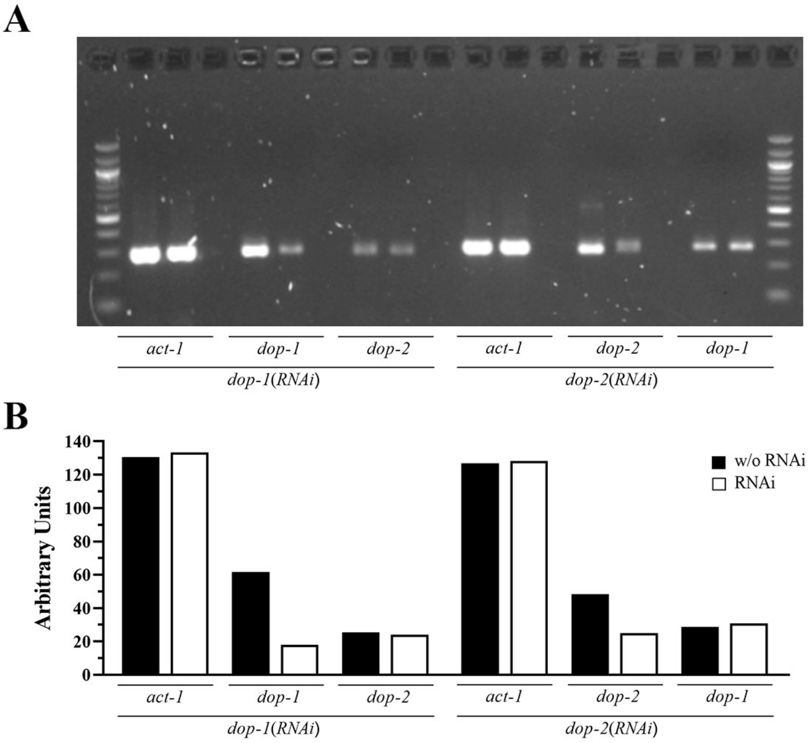

Figure S1. Validation of RNAi efficiency and specificity by semi-quantitative RT-PCR. (**A**) Representative agarose gel showing semi-quantitative RT-PCR amplification of *act-1* (control), *dop-1*, and *dop-2* transcripts in worms subjected to *dop-1*(*RNAi*) or *dop-2*(*RNAi*). Amplification was performed using a limiting number of PCR cycles (28 cycles) to allow detection of differences in transcript levels. Under these conditions, RNAi treatment specifically reduced the expression of the targeted gene, while the expression of non-targeted paralogs remained unchanged. At higher cycle numbers (35 cycles), amplification reached saturation and differences between conditions were no longer detectable (data not shown). **(B)** Densitometric quantification of RT-PCR fragments shown in (A). Intensities were quantified using ImageJ v.1.54 (https://imagej.nih.gov/ij/), background-subtracted, and expressed as arbitrary units.

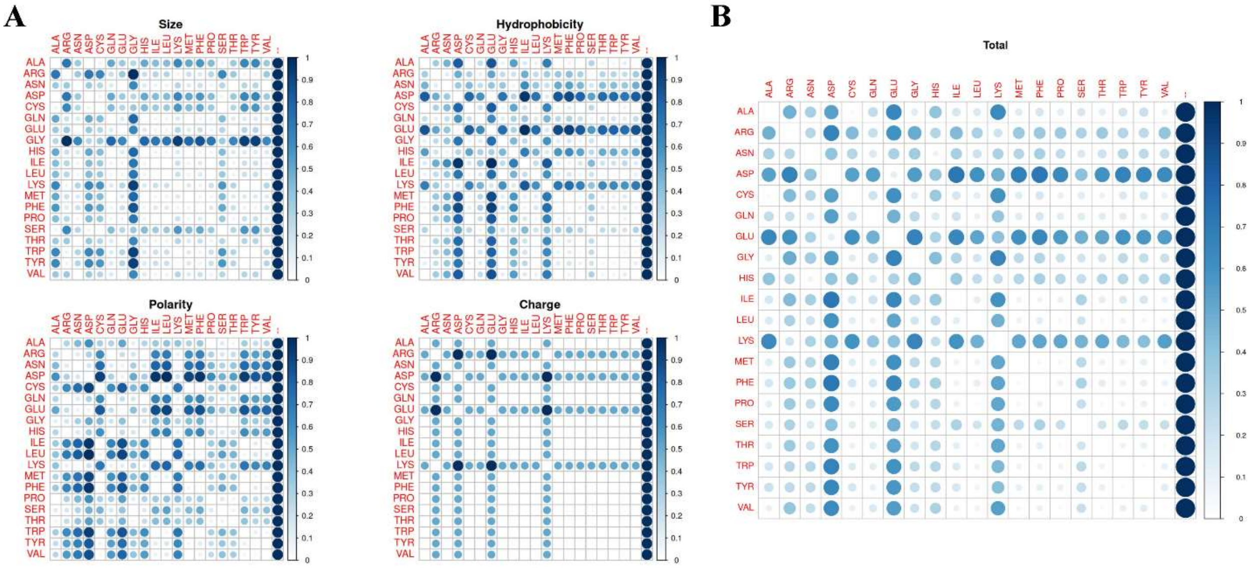

Figure S2. Substitution matrices. (**a**) Normalized substitution matrices for each of the four descriptors (i.e., size, hydrophobicity, polarity, and charge). (**b**) Composite substitution matrix generated by computing the arithmetic mean of the values across the four individual matrices.

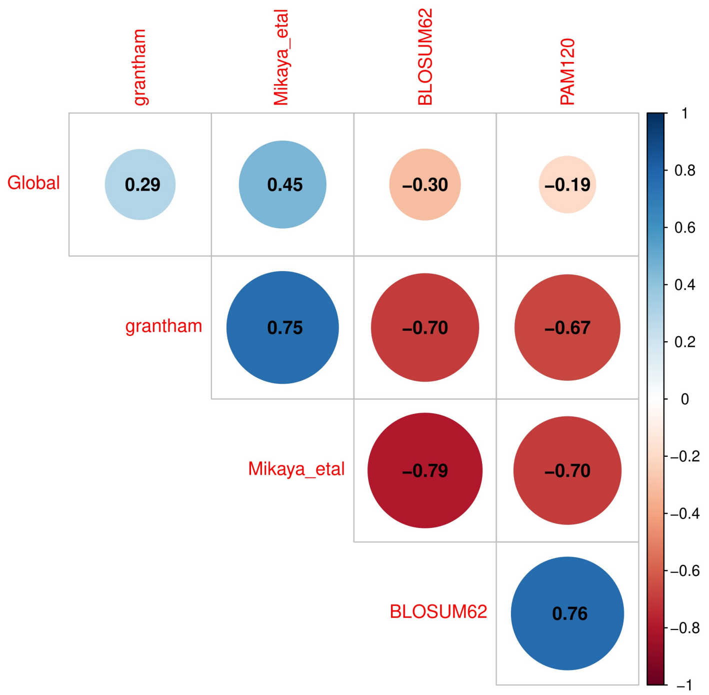

Figure S3. Comparison between substitution matrices. Pearson correlation coefficients computed between our custom matrices and those from the literature.

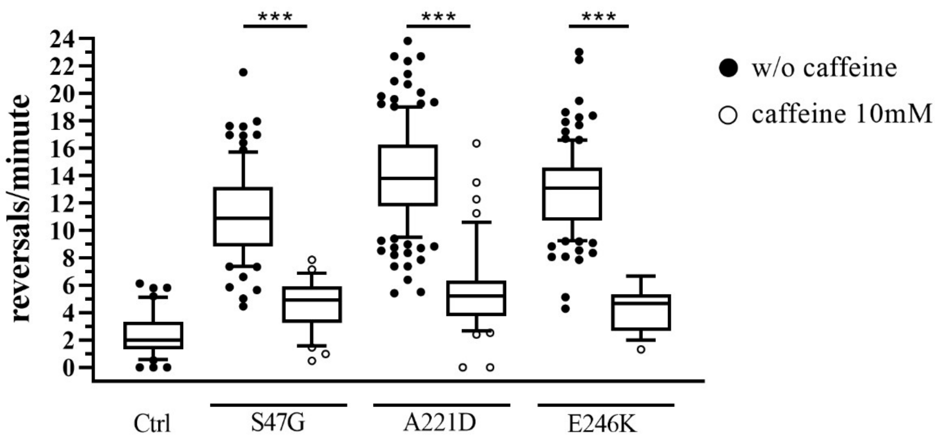

Figure S4. Caffeine suppresses the increased reversal rate in *goa-1* mutants. Reversal rate is expressed as the number of reversals per minute. Data are shown for control (Ctrl) and *goa-1* mutant strains treated or not with caffeine (10 mM, 2 h exposure). Statistical comparisons were performed between treated and untreated animals within each genotype (***$p < 0.0001$; unpaired *t*-test with Welch's correction). Forty animals were analyzed for each genotype and condition.

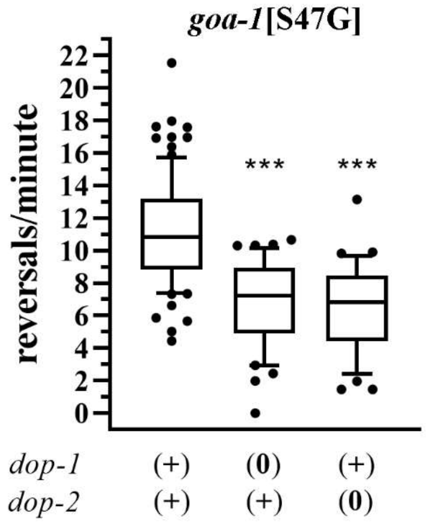

Figure S5. Effect of *dop-1* and *dop-2* loss-of-function on reversal behavior in *goa-1*[S47G] animals. Reversal rate in *goa-1*[S47G] animals carrying loss-of-function (0) mutations in *dop-1* or *dop-2*. Knockout of these genes significantly reduced the hyperactive reversal phenotype of *goa-1*[S47G] animals. However, interpretation of *goa-1*[S47G];*dop-2*(0) animals is limited due to intrinsic locomotor defects, including backward movements, associated with the *dop-2(vs105)* allele [51]. Statistical comparisons were performed between *goa-1*[S47G] and *goa-1*[S47G];*dop-1*(0) animals and between *goa-1*[S47G] and *goa-1*[S47G];*dop-2*(0) animals (***p < 0.0001; unpaired *t*-test with Welch's correction). The number of animals tested is reported in Table S4.

Supplementary Tables

Table S1. Primer pairs and conditions used in RT-PCR assays.

Gene	Forward (5'→ 3')	Reverse (5'→ 3')	Amplicon (bp)	Ann. Temp. (°C)	PCR Cycles
act-1	CAAGAGAGGTATCCTTACCC	AGAGTCGAGGACGACTCCG	286	60	28
dop-1	GCAGTCAACGATATCTTGGG	GACCTGTTAGGTTATTGATGG	283	60	28
dop-2	ATGTATATGTCACCAACGGC	GGTTAGCATCTGGTGGACG	273	58	28

Table S2. Conservation of the AR/caffeine interaction between human and *C. elegans* GPCRs.

A2AR Residues[1]	Distance [Å]	Corresponding *C. elegans* AR Residues	Size Change	Hydrophobicity Change	Polarity Change	Charge Change	Global Change
Phe^{168}	3.246	Phe^{160}	0	0	0	0	0
Ile^{274}	3.307	Val^{263}	0.071	0.139	0.086	0	0.087
Asn^{253}	3.335	Asn^{244}	0	0	0	0	0
Leu^{249}	3.377	Leu^{240}	0	0	0	0	0
Met^{270}	3.585	Trp^{261}	0.143	0.081	0.037	0	0.076
Met^{177}	3.630	Leu^{169}	0.071	0.123	0.099	0	0.08
Val^{84}	3.961	Leu^{83}	0.071	0.099	0.123	0	0.068
Ala^{63}	4.446	Thr^{61}	0.357	0.057	0.062	0	0.118
Glu^{169}	4.535	Glu^{161}	0	0.801	0.457	0.5	0.526
Ile^{66}	4.654	Val^{65}	0.071	0.139	0.086	0	0.087
His^{278}	4.914	His^{267}	0	0	0	0	0

[1]The indicated residues play a key role in caffeine binding, as they contain at least one atom located within 5 Å of the ligand. Residues are ordered according to their distance from caffeine. AR, adenosine receptor; A2AR, adenosine receptor 2A subtype. Substitution scores are normalized to the maximum observed difference for each descriptor.

Table S3. Reversal rate of wild-type (N2) animals exposed to selective agonists or antagonists targeting relevant GPCRs.

Solvent/Compound	Target Receptor	Mechanism of Action	Reversal Rate	*p*-value	N
water			2.71		20
DMSO 0.07%			0.20	0.0121	20
Ethanol 0.2%			2.00	0.5041	20
LE300 (DMSO)	D1R	Antagonist	2.91	0.0010	20
SKF 81297 hydrobromide (DMSO)	D1R	Agonist	0.60	0.2720	20
L-741,626 (ethanol)	D2R	Antagonist	1.10	0.2307	20
Sumanirole maleate (water)	D2R	Agonist	0.90	0.0504	20
AM4113 (ethanol)	CB1R	Antagonist	1.30	0.4838	20
ACEA (ethanol)	CB1R	Agonist	1.10	0.2523	20

Reversal rate is expressed as the number of reversals per minute. N indicates the number of animals analyzed. Statistical comparisons were performed between drug-treated animals and solvent-treated controls using an unpaired t-test with Welch's correction. As previously shown [10], DMSO strongly reduces the reversal rate of N2 animals. D1R, Dopamine D1 subtype receptor; D2R, Dopamine D2 subtype receptor; CB1R, Cannabinoid CB1 subtype receptor.

Table S4. Reversal rate of *goa-1* mutants exposed to selective agonists or antagonists targeting relevant GPCRs.

Compound	Target Receptor	Mechanism of Action	Genetic Background	Reversal Rate	*p*-value	N
			N2	2.4		100
			goa-1[S47G]	11.1	<0.0001	95
			goa-1[A221D]	14.1	<0.0001	154
			goa-1[E246K]	12.9	<0.0001	113
			dop-1(vs100)	1.1	0.0083	22
			dop-2 (vs105)	1.3	0.0176	20
			goa-1[S47G];*dop-1*(0)	6.8	<0.0001	42
			goa-1[S47G];*dop-2*(0)	6.4	<0.0001	38
LE300	D1R	Antagonist				
			goa-1[S47G]	13.3	0.0015	43
			goa-1[A221D]	13.2	0.0924	57
			goa-1[E246K]	12.7	0.6678	28
			goa-1[S47G];*dop-1*(0)	8.4	0.0257	20
SKF 81297 hydrobromide	D1R	Agonist				
			goa-1[S47G]	9.3	0.0019	44
			goa-1[A221D]	11.8	<0.0001	46
			goa-1[E246K]	9.1	0.0003	24
			goa-1[S47G];*dop-1*(0)	8.4	0.0289	26
L-741,626	D2R	Antagonist				
			goa-1[S47G]	7.3	<0.0001	13
			goa-1[A221D]	9.7	<0.0001	21
			goa-1[E246K]	8.9	<0.0001	19
			goa-1[S47G];*dop-2*(0)	5.9	0.3349	25
Sumanirole maleate	D2R	Agonist				
			goa-1[S47G]	9.5	0.0427	22
			goa-1[A221D]	13.4	0.3687	16
			goa-1[E246K]	10.6	0.0476	12
			goa-1[S47G];*dop-2*(0)	7.4	0.1832	28
AM4113	CB1R	Antagonist				
			goa-1[S47G]	8.8	0.0011	29
			goa-1[A221D]	8.4	0.0011	20
			goa-1[E246K]	11.3	0.0014	35
ACEA	CB1R	Agonist				
			goa-1[S47G]	12.1	0.1134	46
			goa-1[A221D]	12.4	0.0028	45
			goa-1[E246K]	15.1	0.0091	24

Reversal rate is expressed as the number of reversals per minute. N indicates the number of animals scored. Comparisons between *goa-1* mutants and isogenic control animals and comparisons for each *goa-1* mutant strain treated or not with the indicated drug were performed using unpaired t-test with Welch's correction. D1R, Dopamine D1 subtype receptor; D2R, Dopamine D2 subtype receptor; CB1R, Cannabinoid CB1 subtype receptor.

Table S5. Forward locomotion speed of the *goa-1*[R209H] mutant exposed to selective agonists or antagonists targeting relevant GPCRs.

Compound	Target Receptor	Mechanism of Action	Genetic Background	Speed	*p*-value	N
			N2	30.1		100
			goa-1[R209H]	87.7	<0.0001	40
Ethanol 0.2%			*goa-1*[R209H]	111.4	<0.0001	87
LE300	D1R	Antagonist				
			goa-1[R209H]	100.5	0.0099	35
SKF 81297 hydrobromide	D1R	Agonist				
			goa-1[R209H]	103.6	0.0020	35
L-741,626	D2R	Antagonist				
			goa-1[R209H]	93.6	0.0498	19
Sumanirole maleate	D2R	Agonist				
			goa-1[R209H]	102.1	0.0004	28
AM4113	CB1R	Antagonist				
			goa-1[R209H]	93.3	<0.0001	80
ACEA	CB1R	Agonist				
			goa-1[R209H]	136.8	<0.0001	35

Speed is expressed in µm per second. N indicates the number of animals scored. Speed after 2 h exposure to the selected compound was compared with that observed following 2 h exposure to the corresponding control, defined by the solvent used to dissolve the compound. The speed of *goa-1*[R209H] animals was also compared to that of isogenic control worms. Unpaired t-test with Welch's correction was used for statistical analyses. D1R, Dopamine D1 subtype receptor; D2R, Dopamine D2 subtype receptor; CB1R, Cannabinoid CB1 subtype receptor.

Table S6. Reversal rate in *C. elegans* strains following RNAi-mediated knockdown of genes encoding GPCRs acting upstream of inhibitory or stimulatory G proteins.

Genetic Background	Target Gene	Reversal Rate	Embryonic lethality (%)	*p*-value	N
rrf-3(0)					
	ama-1	-	100	<0.0001	200
	EV	1.16			30
	dop-2	1.99		0.0184	43
	dop-3	1.32		0.6208	33
	gbb-1	1.81		0.0729	31
	gbb-2	1.48		0.4218	36
	npr-19	1.33		0.5745	41
	dop-1	1.83		0.7390	16
	ser-1	2.62		0.1045	15
	mgl-2	1.50		0.6736	16
rrf-3(0);*goa-1*(0)					
	EV	9.64			11
	dop-1	6.88		0.0096	20
	ser-1	6.90		0.0049	20
	mgl-2	7.13		0.0114	20
rrf-3(0);*goa-1*[S47G]					
	EV	8.27			10
	dop-1	5.40		0.0014	20
	ser-1	6.20		0.0086	20
	mgl-2	5.53		0.0011	20
rrf-3(0);*goa-1*[E246K]					
	EV	9.72			10
	dop-1	8.49		0.2255	18
	ser-1	8.67		0.2795	14
	mgl-2	7.95		0.0861	16
	EV	7.51			60
	dop-2	6.52		0.1153	45
	dop-3	6.47		0.1195	32
	npr-19	6.76		0.6142	25

Reversal rate is expressed as the number of reversals per minute. N indicates the number of animals scored. In each genetic background, the reversal rate measured following RNAi-mediated knockdown of genes encoding GPCRs that act upstream to inhibitory (*dop-2*, *dop-3*, *gbb-1*, *gbb-2*, and *npr-19*) or stimulatory (*dop-1*, *ser-1*, and *mgl-2*) G proteins was compared with that observed in lines treated with the empty vector (EV), using unpaired t-test with Welch's correction. *ama-1 (RNAi)* was used as a positive control to determine the efficiency of RNA interference.

Table S7. Forward locomotion speed measured in *rrf-3*(0);*goa-1*[R209H] animals following RNAi-mediated knockdown of genes encoding GCPRs acting upstream to stimulatory G proteins.

Genetic Background	Target Gene	Speed	Embryonic lethality (%)	*p*-value	N
rrf-3(0);*goa-1*[R209H]					
	ama-1	-	100	<0.0001	200
	EV	85.94			14
	dop-1	76.63		0.0499	28
	ser-1	69.21		0.0415	17
	mgl-2	64.08		0.0347	10

Speed is expressed in µm per second. N indicates the number of animals scored. Speed after RNAi-mediated knockdown of genes encoding GPCRs that act upstream of stimulatory (*dop-1*, *ser-1*, *mgl-2*) G proteins was compared with those observed in lines treated with the empty vector (EV), using unpaired t-test with Welch's correction. *ama-1(RNAi)* was used as a positive control to determine the efficiency of RNA interference.

Table S8. Reversal rate in *goa-1*(0) animals crossed with lines knockout for genes encoding GPCRs acting upstream to stimulatory G proteins.

Genetic Background	Reversal Rate	*p*-value	N
goa-1(0)	9.28		80
goa-1(0);*dop-1*(0)	7.67	<0.0001	96
goa-1(0);*ser-1*(0)	7.14	<0.0001	49
goa-1(0);*mgl-2*(0)	7.01	<0.0002	37

Reversal rate is expressed as the number of reversals per minute. N indicates the number of animals scored. Values of *goa-1*(0) animals were compared with those of double mutants, using unpaired t-test with Welch's correction.
